# Dynamic Regulation of Phenylalanine Hydroxylase by Simulated Redox Manipulation

**DOI:** 10.1371/journal.pone.0053005

**Published:** 2012-12-31

**Authors:** Julian E. Fuchs, Roland G. Huber, Susanne von Grafenstein, Hannes G. Wallnoefer, Gudrun M. Spitzer, Dietmar Fuchs, Klaus R. Liedl

**Affiliations:** 1 Institute of General, Inorganic and Theoretical Chemistry, and Center for Molecular Biosciences Innsbruck (CMBI), University of Innsbruck, Innsbruck, Austria; 2 Division of Biological Chemistry, Biocenter, Innsbruck Medical University, Innsbruck, Austria; Oak Ridge National Laboratory, United States of America

## Abstract

Recent clinical studies revealed increased phenylalanine levels and phenylalanine to tyrosine ratios in patients suffering from infection, inflammation and general immune activity. These data implicated down-regulation of activity of phenylalanine hydroxylase by oxidative stress upon *in vivo* immune activation. Though the structural damage of oxidative stress is expected to be comparably small, a structural rationale for this experimental finding was lacking. Hence, we investigated the impact of side chain oxidation at two vicinal cysteine residues on local conformational flexibility in the protein by comparative molecular dynamics simulations. Analysis of backbone dynamics revealed a highly flexible loop region (Tyr138-loop) in proximity to the active center of phenylalanine hydroxylase. We observed elevated loop dynamics in connection with a loop movement towards the active site in the oxidized state, thereby partially blocking access for the substrate phenylalanine. These findings were confirmed by extensive replica exchange molecular dynamics simulations and serve as a first structural explanation for decreased enzyme turnover in situations of oxidative stress.

## Introduction

The mononuclear non-heme iron containing monooxygenase phenylalanine hydroxylase (PAH, EC 1.14.16.1) is a member of the aromatic amino acid hydroxylase family as tyrosine hydroxylase or tryptophan hydroxylases 1, 2. It catalyzes the oxidation of phenylalanine (Phe) to tyrosine (Tyr). This oxidation of the substrate amino acid is achieved by molecular oxygen and the reductive co-factor 5,6,7,8-tetrahydrobiopterin (BH_4_) [1]. Hydroxylation of Phe to Tyr is the committed step in Phe catabolism and therefore requires strict regulation to ensure homeostasis of the essential amino acid Phe. Dysfunction of PAH causes phenylketonuria (PKU) [2], [3], a common and well-examined genetic disease [4] leading to mental retardation upon accumulation of Phe [5].

The catalytic mechanism of PAH involves the reduction from ferric (III) to ferrous (II) form by the co-factor BH_4_. Ferrous iron is subsequently oxidized by molecular oxygen to a Fe(IV)O intermediate, which in turn hydroxylates Phe to Tyr [6]–[9]. Eukaryotic PAH is found as a homotetramer, where each subunit contains three distinct domains: an N-terminal autoregulatory domain (residues 1–142), the catalytic domain (residues 143–410) and a tetramerization domain stabilizing the quaternary structure (residues 411–452) [10], [11]. BH_4_ acts as a negative regulator of PAH activity by stabilizing an inactive form [12], whereas the enzyme is activated by phosphorylation of Ser16 [13] as well as binding of Phe [14]. Activity of PAH increases upon preincubation with Phe up to 100-fold [15]. Allosteric effects are held responsible for the activating effect of Phe by conformational changes in the protein [16]. The positive cooperativity of Phe binding allows for fast responses to increased Phe levels in order to avoid damages to the brain [17]. Recently, a potential binding site of Phe distal to the active site was discovered by a crystallographic study of PAH from *Chromobacterium violaceum* [18].

Expression of the truncated PAH catalytic domain does not result in a loss in activity suggesting the autoregulatory domain to be a key factor in allosteric regulation [19]. The structural background of PAH activation remains still unclear, as crystal structures are not yet available for the full sequence protein. The only available crystal structures containing the autoregulatory domain of a dimeric rat PAH lack parts of the tetramerization domain and structural insights into residues 1–18 including the phosphorylation site at Ser16 [20], [21]. Nevertheless, the position of the additional residues of the regulatory domain suggest a complete physical blockage of the active site in the inactive enzyme [22]. However, it remains unclear, if activation is due to a localized movement of the regulatory sequence or a global conformational reorganization in the protein.

Current studies found immune activation and inflammation to be associated with an increase in the ratio of Phe to Tyr in blood of patients suffering from trauma, sepsis, carcinoma and infection [23]–[25] suggesting an inhibition of PAH [26]. Recently, treatment with immune modulating interferon-α of patients with hepatitis C virus, resulted in an increase of Phe and the Phe to Tyr ratio [27] consistent with earlier findings concerning patients with multiple melanoma treated with interferon-α [28]. As immune activation of macrophages is paralleled by the release of toxic reactive oxygen species (ROS) and neopterin [29], [30], oxidative stress is discussed as chemical background for PAH deactivation consistent with studies correlating PKU with oxidative stress [31]. This hypothesis is further strengthened, as phenylalanine concentrations in blood samples of cancer patients were shown to correlate to the oxidative stress marker isoprostane-8 [24]. Immune activation marker neopterin was shown to further increase oxidizing capacity of ROS [32] and therefore raise concentrations of protein oxidation products [33] whilst decreasing antioxidant levels [34]. Modulation of PAH activity has also been reported after reaction with various disulfide reagents [35], [36], pointing particular attention to cysteine residues in PAH.

**Figure 1:**
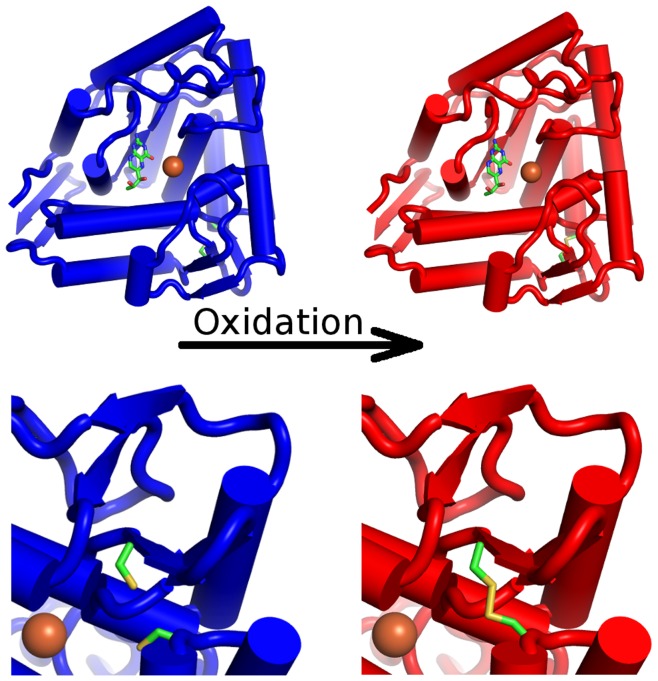
Structural overview of native and oxidized phenylalanine hydroxylase. The catalytic domain of native phenylalanine hydroxylase in blue cartoon representation (top left). The biopterin co-factor in the active site is shown as sticks, the catalytic iron as brown sphere. Cysteine residues 203 and 334 are shown as sticks to highlight the artificially introduced oxidation site distant from the catalytic center (red, top right). A zoom at the site of oxidation at the back of the top figures is shown at the bottom. Cys203 and Cys334 in proximity to each other (native state, blue, bottom left) are closed to a disulfide bond (oxidized state, red, bottom right).

To investigate the impact of oxidative stress on PAH structure and dynamics at atomic level, molecular dynamics (MD) simulations of the catalytic domain were performed. Inspection of 23 available X-ray structures of the catalytic domain of PAH in the PDB [37], [38] revealed one single apparent oxidative site at two free cysteine residues (Cys203, Cys334) in close proximity to each other (4 Å) but distant from the active center of the enzyme (15 Å) (see Figure 1). An intrinsic connection between these cysteine residues and PAH activity *in vivo* is documented by mutation data from genetic studies of PKU. Point mutations of both residues are associated with mild forms of PKU [39]–[41].

Comparative MD simulations were performed to analyze conformational differences between native PAH (PAH_nat_) and PAH in oxidized state (PAH_ox_) containing an artificially introduced disulfide bond between these residues. Findings from these simulations were confirmed by follow-up replica exchange molecular dynamics (REMD) simulations increasing sampling of the observed conformational transitions in the protein.

## Methods

Two MD simulations were set up in parallel using the aforementioned starting structures PAH_nat_ and PAH_ox_. In simulation PAH_nat_ of native PAH we treated Cys203 and Cys334 as individual cysteine residues, as reported in all available X-ray structures. In simulation of PAH_ox_ these cysteine residues were connected via an introduced disulfide bond to represent an oxidized state of PAH. This introduced disulfide bond between Cys203 and Cys334 is the only difference between the two simulated states PAH_nat_ and PAH_ox_.

As a starting point for simulations we chose the X-ray structure of human PAH with bound co-factor and highest available resolution (PDB: 1J8U [42], resolution:1.50 Å), although the bound co-factor and the iron atom were both found in reduced form. All atom root mean square deviation (RMSD) values to high resolution X-ray structures containing the oxidized species (PDB: 1TG2 [43], 1LTZ [44]) were found to be reasonably low (RMSD <1.13 Å) to set up simulations using template structure 1J8U with both oxidized co-factor 7,8-dihydrobiopterin BH_2_ and Fe(III).

Both all-atom MD simulations of the PAH catalytic domain containing a Fe(III)-ion and the co-factor BH_2_ were carried out using the AMBER10 package [45] using the ff99SB parameter set [46] for the protein and GAFF parameters [47] for the co-factor. Partial charges for the co-factor were derived by RESP fitting [48] at HF-6/31G*-level using Gaussian03 [49]. Van der Waals parameters for Fe(III) were derived by distance scanning against a helium atom at HF-6/31G*-level and found to be r = 1.20 Å and e = 0.0140 kcal/(mol*cm) in agreement with other transition metals in ff99SB.

Glutamate as well as aspartate residues were deprotonated, lysine and arginine residues protonated. Histidine (His) protonation was carried out manually according to the respective chemical environment: His200, His201, His208 and His271 at the protein surface were protonated at both imidazole nitrogens, His146, His170, His264 were assigned ε-hydrogen atoms. His285 and His290 coordinating the Fe(III)-ion at the active center were assigned δ-hydrogen atoms. The total charge of the system was found to be +3, which was neutralized by a uniform neutralizing plasma for Particle Mesh Ewald simulations [50].

The solvent was treated explicitly in the simulations including all resolved water molecules from the X-ray structure. The system was soaked with a wall distance of 10 Å by superposing an octahedral box of 7554 TIP3P [51] water molecules, resulting in a box edge of 70.8 Å after NpT equilibration.

After relaxation with harmonic restraints on heavy protein atoms and subsequent minimization, the systems were heated gradually from 100 K to 300 K over 200 ps in NVT ensemble. After heating and 1 ns of pressure and density equilibration, a NpT MD production run of 200 ns was performed to allow for larger conformational rearrangement in the enzyme. Simulations were carried out at 300 K using a Langevin thermostat [52] at 1.0 bar pressure with 8.0 Å nonbonded cutoff. SHAKE algorithm [53] on hydrogen atoms allowed a 2 fs time step. Snapshots were saved to trajectory every 500 steps or equivalent 1 ps for further analysis.

REMD simulations increase conformational sampling compared to standard MD simulations by exchanging replica of the simulated system at different temperatures, if an energy criterion is met. Hence, energy barriers are more likely to be overcome extending the sampling of conformational space [54]. In this study, equilibrated systems of standard MD simulations were used as input coordinates for two REMD simulations (PAH_nat_, PAH_ox_). Sixteen parallel simulations were performed for both systems in a temperature spacing of 2 K as suggested by Patriksson et al [55] in a temperature range from 300 to 330 K. Exchange probability was set to 0.25 whilst attempting 10 exchanges per nanosecond. Besides a change to NVT ensemble, which is justified after volume adaption during pressure equilibration, identical parameters as for standard MD simulations were applied for REMD simulations. Production runs were performed for the two systems at 16 temperatures for 100 ns, resulting in total REMD trajectory length of 1.6 µs each.

Trajectories were analyzed using ptraj (version 4/2010) from AmberTools [45]. Positional fluctuations were calculated as RMSD of Cα-atoms to assess stability of the simulations. B-factors (temperature factors) were calculated residue-wise as a measure of individual local flexibility. Structures were visualized using pymol [56]. This software package was also used to calculate the accessible surface area of the catalytic Fe(III)-ion over the simulation time by the command ‘get_area’. Binding site volumes were estimated using POVME [57] by creating a 6 Å sphere around the center Fe(III)-ion filling the active site cavity with 1 Å spaced grid points. Subsequently, grid points near protein atoms were removed yielding an estimate of the actual binding site by summing up volume elements of residual grid points.

## Results

**Figure 2:**
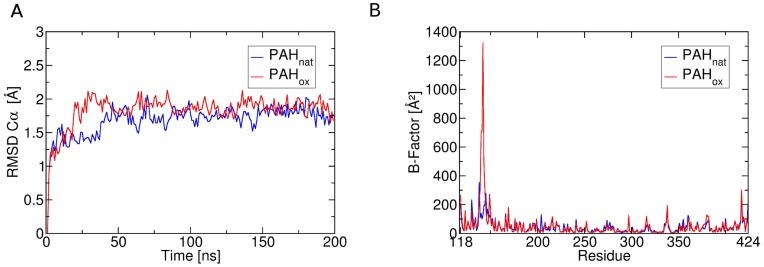
Global dynamic behavior of native and oxidized phenylalanine hydroxylase. A: RMSD of Cα-atoms over the simulation time of 200 ns standard MD simulations for the two systems PAH_nat_ (blue) and PAH_ox_ (red). After an initial phase of loop reorientation for the oxidized system PAH_ox_ (around 20 ns simulation time), simulations yield stable trajectories. B: Residue-wise B-factors for the simulated systems highlight residues 130–145 as particularly altered by the introduced cysteine oxidation. Elevated B-factors in this loop region (Tyr138-loop) in PAH_ox_ are caused by a reorientation of the loop in an early stage of the simulation. Dynamics of residues around the oxidation sites (Cys-203, Cys334) are similar in both systems.

After equilibration, for both simulated systems stable molecular dynamics trajectories over 200 ns with mean backbone Cα RMSD values below 2 Å could be generated (see Figure 2). After 20 ns simulation PAH_ox_ shows a sharp increase in backbone dynamics as a loop region around residue 140 distant from the artificially linked cysteine residues starts to rearrange. Despite this structural transition in the so-called Tyr138-loop [58]–[60], simulation PAH_ox_ yields a stable trajectory for the whole simulated system resulting in an RMSD plateau below 2 Å at comparable level to simulation PAH_nat_.

Calculated B-factors also highlight the Tyr138-loop as region of particular interest with B-factors reaching beyond 1000 Å^2^ in PAH_ox_ compared to 300 Å^2^ in PAH_nat_. Other regions show less pronounced flexibility and similar behavior in both MD simulations. This finding also holds true for the vicinities of Cys203 and Cys334, where the artificial oxidation was introduced in PAH_ox_, which are found to be rigid in both simulations.

**Figure 3:**
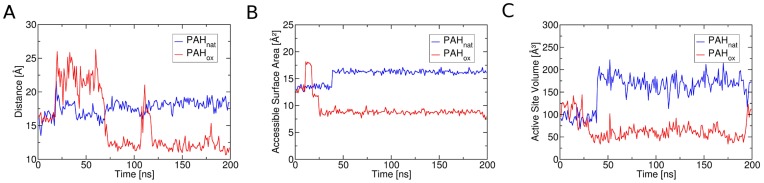
Dynamics of the binding site region of native and oxidized phenylalanine hydroxylase. A: Distance between the catalytic iron center and the Cα-atom of Glu141 included in the flexible Tyr138-loop near the active site. Whereas this distance remains mostly stable in the native state PAH_nat_ over the simulation time (blue), simulation of PAH_ox_ shows an initial phase of increased loop flexibility that is followed by decrease of that distance indicating a loop movement towards the active site. This finding is confirmed by calculation of the accessible surface area of the iron center (B). After an initial adaption to the perturbation, PAH_ox_ shows a constantly reduced accessibility of the catalytic iron center compared to PAH_nat_. The overall active site volume (C) is similarly reduced over simulation time in PAH_ox_ compared to PAH_nat_, suggesting a reduced enzyme turnover.

As indicated by the distance plot in Figure 3, the flexible Tyr138-loop performs a movement towards the active site of the protein after an initial phase of elevated dynamics in PAH_ox_ (15–70 ns) leading to a contraction of the binding site. This movement is not present in the simulation of the native state PAH_nat_. This movement in PAH_ox_ is paralleled by a decrease in accessibility of the catalytic iron center indicated by lowered accessible surface area of below 10 Å^2^ in PAH_ox_ compared to over 15 Å^2^ in PAH_nat_ (see Figure 3 and Table 1). Furthermore, the total binding site volume is reduced significantly in the course of this loop rearrangement in PAH_ox_: An average active site volume of over 150 Å^3^ in PAH_nat_ is reduced to less than half in PAH_ox_ by the introduced perturbation.

**Table 1:** Averages and standard deviations (SD) of accessible surface area of the catalytic iron as well as the total active site volume for the simulations of PAH_nat_ and PAH_ox_.

	200 ns MD		16*100 ns REMD	
Average (SD)	PAH_nat_	PAH_ox_	PAH_nat_	PAH_ox_
Area [Å^2^]	15.74 (1.16)	9.40 (1.97)	15.38 (2.33)	13.68 (3.07)
Volume [Å^3^]	155.87 (35.11)	66.58 (22.73)	116.15 (54.42)	104.76 (45.82)

REMD simulations confirm the reduction in both measures for PAH_ox_ in comparison to PAH_nat_ for an average over 16 simulations over 100 ns compare to a single observation in a 200 ns standard MD run.

REMD simulations yielded comparably stable trajectories (see Figure S1), confirming trends observed from for standard MD simulations. B-factors in the Tyr138-loop are generally elevated in PAH_ox_ compared to PAH_nat_ (see Figure S2). This observation is true for every single direct comparison of 16 temperature-equivalent replicas. Likewise, PAH_ox_ shows a lowered average accessibility of the catalytic iron of 13.7 Å^2^ compared to 15.4 Å^2^ in PAH_nat_. The volume of the active site is decreased similarly in the oxidized state from 116.2 Å^3^ in PAH_nat_ to 104.8 Å^3^ in PAH_ox_.

## Discussion

As a structural explanation of the impairment of PAH caused by oxidative stress has been lacking by now, we propose a computational model of an oxidized PAH specie. This protein structure shows an altered behavior in MD simulations in the Tyr138-loop in close proximity to the active site [61] leading to a stable structure showing a contracted binding site. As the loop rearrangement occurs as a single event in the 200 ns MD trajectory, the statistical significance of this observation cannot be inferred from standard MD simulations of that time scale [62]. This intramolecular movement hence appears on a time scale beyond sampling of standard state-of-the-art MD simulations.

To statistically substantiate this proposed structural rationale for PAH down-regulation, REMD simulations providing improved conformational sampling were performed. These computationally demanding simulations agreed with trends seen in standard MD simulations, namely elevated loop dynamics paralleled with a decrease in accessibility of the catalytic iron center and binding site contraction. This contraction of the binding pocket would obviously lead to reduced substrate binding and hence enzyme down-regulation or even dysfunction. The enhanced sampling by REMD simulations decreased the observed differences between the simulations of PAH_nat_ and PAH_ox_, but still supported findings from standard MD simulations, as 16 additional parallel simulations confirmed the trends observed from a single standard MD run.

Findings from MD simulations linking dynamics of the Tyr138-loop region with catalytic activity are in best agreement with experimental data on allosteric regulation of PAH. Li and co-workers showed by an increase in exchange kinetics of hydrogen/deuterium exchange mass spectrometry, that region 129–143 is indeed a solvent-accessible and highly flexible region in PAH that is influenced by presence of Phe [63]. This observation is further confirmed, as this region is not resolved in several crystal structures of PAH in absence of a substrate stabilizing the active site. Furthermore, modulation of the dynamics of region 129–143 by incubation with phenylalanine, an activator of PAH, indicates a mechanistic role in allosteric regulation [63].

**Figure 4:**
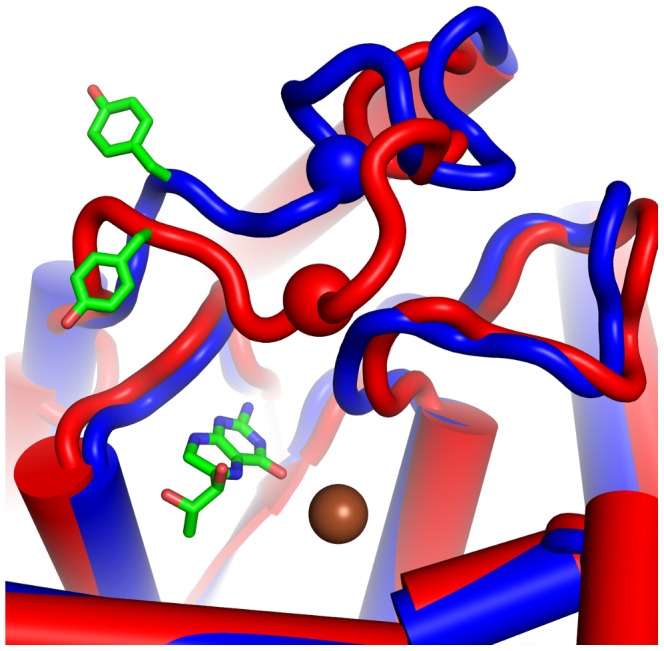
Changes in the active site of phenylalanine hydroxylase introduced by side chain oxidation. Starting coordinates of both simulations (blue cartoon) overlaid with an average structure of the time frames 150–160 ns of standard MD simulation PAH_ox_ (red cartoon). The pronounced loop movement towards the catalytic center (iron ion as brown sphere, co-factor as sticks) was measured as distance of the Cα-atom of Glu141 (red and blue sphere respectively) to the catalytic iron (brown). This rearrangement is paralleled by a reorientation of Tyr138 (shown as sticks, left), in turn concertedly blocking access to the active site of PAH_ox_.

The critical regulatory role of surface loops has been discussed in a recent review on allosteric regulation of PAH by Fitzpatrick [60]. He describes the Tyr138-loop as undergoing the most drastic change upon ligand binding in accordance with crystal structure analyses of Andersen et al [58], [59]. Tyr138 included in that loop closes down on the active site leading to a major contraction restricting further access similar to the movement observed in our simulations (see Figure 4). A similar movement of a surface loop has been proposed by Sura et al. for the homologous enzyme tyrosine hydroxylase [64].

This dynamic behavior of the loop region could be seen as an analogy to the behaviour of the autoregulatory sequence of PAH. This domain of the protein physically blocks entrance to the active site of PAH in the inactive form. A smaller but similar impact of the surface loop can be inferred from presented molecular dynamics simulations directly explaining the observed PAH impairment upon oxidative stress.

The importance of conformational flexibility for PAH activity has been revealed in several studies: Cerreto et al and Gersting et al showed correlations between PAH conformational stability and PAH down-regulation by PKU-causing mutations [65], [66]. Changes in stability were shown by MD simulations upon phosphorylation of Ser-16 [67]. This computational technique was also used to infer factors for specificity and affinity of PAH ligand binding [68], [69].

**Figure 5:**
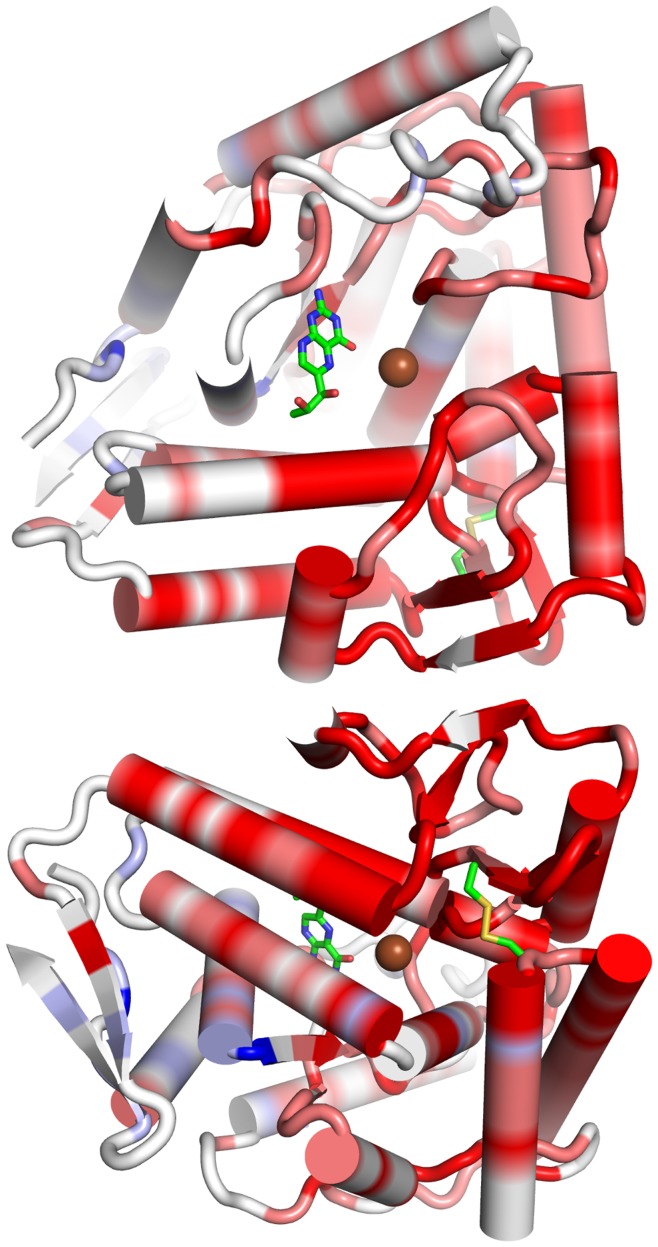
Alteration in local dynamics in phenylalanine hydroxylase upon side chain oxidation. Residue-wise B-factors for the 2*16 REMD trajectories were calculated for Cα-atoms and compared between simulations of equal temperatures. Rank-based differences were mapped on the protein structure, where blue regions indicate regions more flexible in more of the 16 simulations of PAH_nat_. Unaffected regions are shown in white, whereas red regions indicate regions showing elevated backbone dynamics in more of the 16 simulations of PAH_ox_. Elevated B-factors in the central helices suggest an allosteric signal transduction over this path from the introduced oxidation site (Cys203, Cys334) to the Tyr138-loop near the catalytic center. The bottom picture shows the same structure rotated by 220° to highlight local effects on the oxidation site in the back of the top picture.

As presented MD simulations allow to keep track of alterations in molecular motions within the protein, a pathway for the inferred allosteric signaling between Cys203/Cys334 and the active site could be anticipated. An analysis of hydrogen bonding within the protein did not highlight a particular alteration in molecular interactions within the protein but rather suggested a concerted flow of dynamic behavior throughout the protein backbone of PAH (see Figure 5). The differences in molecular flexibility between PAH_ox_ and PAH_nat_ REMD simulations are highlighted by a color-coding from blue for regions more flexible in PAH_nat_ over white to red for regions more flexible in PAH_ox_. The dominating red coloring of PAH_ox_ starting from the artificial oxidation site propagates over the protein backbone towards the surface loops in close proximity to the active site, whereas only minor blue regions are found in a small part of the binding site next to the biopterin co-factor. This highlights a putative interaction path for the allosteric effect of disulfide formation on the active center via the central helices directly to the Tyr138-loop near the active site. A loop movement near the active site in turn restricts accessibility of the catalytic site, hence explaining reduced enzyme turnover.

Our study offers a new example in the emerging field of redox signaling in enzyme activity, that is facilitated by novel findings concerning molecular mechanisms of redox switches in proteins [70]. Schroeder et al for example recently highlighted how the reduction of disulfide bonds activates an antimicrobial protein [71]. Tanner et al. recently reviewed the redox regulation of protein tyrosine phosphatases, where an active site cysteine takes several oxidation states and is inactivated by redox signaling [72]. A regulatory mechanism similar to the proposed signal propagation in PAH has been found by Wang et al. in C-reactive protein, where a single disulfide bond serves as a switch for the function of the protein [73].

An obvious limitation of the presented study is the underlying simplistic model of PAH_ox_. Although reduced Cys203 and Cys334 in close proximity are a clear weak-point for oxidation in the protein, this model might not cover all effects of oxidative stress. Cysteine residues might undergo further oxidation processes besides simple disulfide formation. On the other hand, also the active site of PAH is susceptible to oxidative species, as the enzyme co-factor is readily oxidizable under oxidizing conditions. These latter two effects could not be covered in the presented simulations and hence might be starting points for further investigations from theoretical and experimental side.

In conclusion, our study provides a structural basis for the down-regulation of PAH activity upon oxidative stress. MD simulations highlight a flexible loop region around residue 140 (Tyr138-loop) as susceptible to side chain oxidation in PAH. A complex movement over the whole protein leads to a contraction of the binding site leading to the experimentally observed reduced enzyme activity.

## Supporting Information

Figure S1 **Dynamics of REMD simulations.** A: RMSD of Cα-atoms over the simulation time of 16 REMD simulations a 100 ns of PAH_nat_ (blue). Stable trajectories showing comparable deviations to the starting structure as in standard molecular dynamics runs were obtained. The same holds true for 16 REMD simulations of PAH_ox_ (red, B), where deviations are slightly elevated due to the initial perturbation by disulfide formation.
(TIFF)

Figure S2 **Positional fluctuations in REMD simulations.** A: Residue-wise B-factors for 16 REMD simulations a 100 ns of PAH_nat_ (blue). The flexible Tyr138-loop shows several conformational transitions, whereas other parts of the protein remain stable. The Tyr138-loop shows even higher elevated B-factors in PAH_ox_ (red, B) indicating a major rearrangement in this loop region.
(TIFF)
