# Supplementary figures and images for: Dynamic Regulation of Phenylalanine Hydroxylase by Simulated Redox Manipulation
Source: PLoS One. 2012 Dec 31;7(12):e53005. doi: 10.1371/journal.pone.0053005 (PMC3534100; doi:10.1371/journal.pone.0053005)

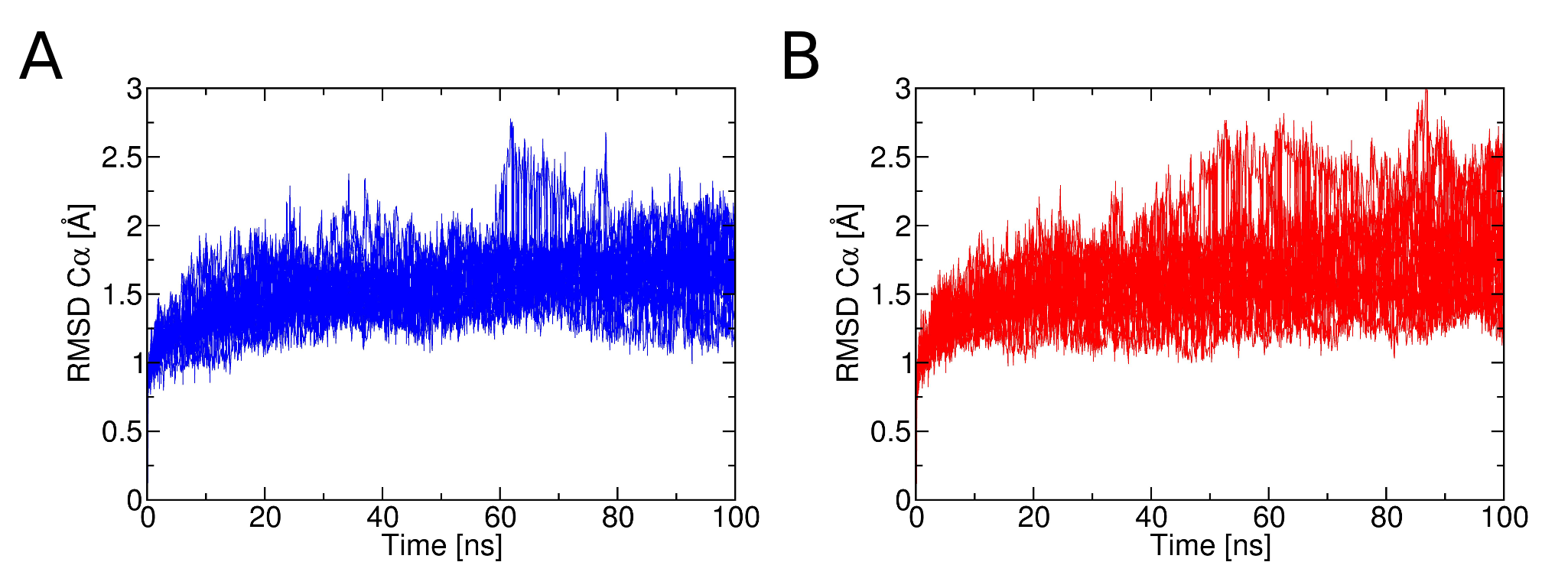

Supplement: Figure S1 — Dynamics of REMD simulations. A: RMSD of Cα-atoms over the simulation time of 16 REMD simulations a 100 ns of PAHnat (blue). Stable trajectories showing comparable deviations to the starting structure as in standard molecular dynamics runs were obtained. The same holds true for 16 REMD simulations of PAHox (red, B), where deviations are slightly elevated due to the initial perturbation by disulfide formation. (TIFF) [file pone.0053005.s001.tiff]

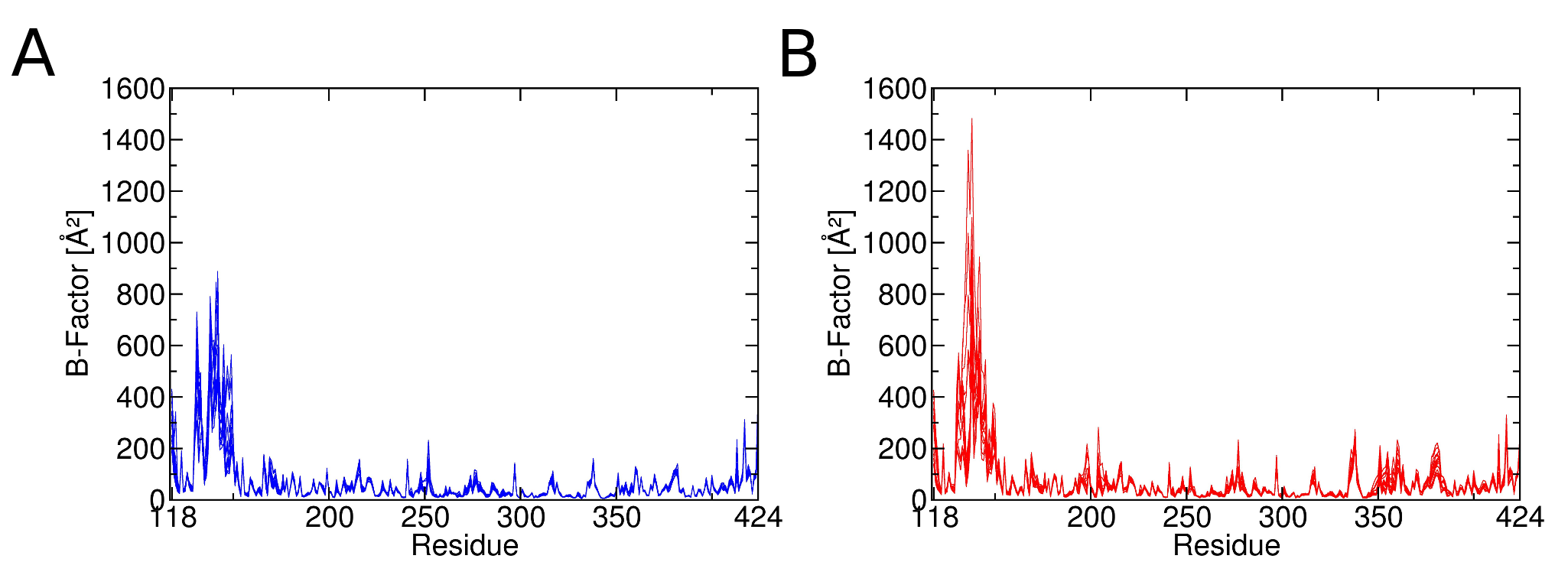

Supplement: Figure S2 — Positional fluctuations in REMD simulations. A: Residue-wise B-factors for 16 REMD simulations a 100 ns of PAHnat (blue). The flexible Tyr138-loop shows several conformational transitions, whereas other parts of the protein remain stable. The Tyr138-loop shows even higher elevated B-factors in PAHox (red, B) indicating a major rearrangement in this loop region. (TIFF) [file pone.0053005.s002.tiff]
